# Exploring How Low Oxygen Post Conditioning Improves Stroke-Induced Cognitive Impairment: A Consideration of Amyloid-Beta Loading and Other Mechanisms

**DOI:** 10.3389/fneur.2021.585189

**Published:** 2021-03-24

**Authors:** Zidan Zhao, Rebecca J. Hood, Lin Kooi Ong, Giovanni Pietrogrande, Sonia Sanchez Bezanilla, Kirby E. Warren, Marina Ilicic, Murielle G. Kluge, Clifford TeBay, Ole P. Ottersen, Sarah J. Johnson, Michael Nilsson, Frederick R. Walker

**Affiliations:** ^1^School of Biomedical Sciences and Pharmacy, University of Newcastle, Newcastle, NSW, Australia; ^2^Priority Research Centre for Stroke and Brain Injury, University of Newcastle, Newcastle, NSW, Australia; ^3^Hunter Medical Research Institute, Newcastle, NSW, Australia; ^4^National Health and Medical Research Council Centre of Research Excellence in Stroke Rehabilitation and Brain Recovery, Heidelberg, VIC, Australia; ^5^School of Pharmacy, Monash University Malaysia, Bandar Sunway, Malaysia; ^6^Division of Anatomy, Department of Molecular Medicine, Institute of Basic Medical Sciences, University of Oslo, Oslo, Norway; ^7^Office of the President, Karolinska Institutet, Stockholm, Sweden; ^8^School of Electrical Engineering and Computing, University of Newcastle, Newcastle, NSW, Australia; ^9^Centre for Rehab Innovations, University of Newcastle, Newcastle, NSW, Australia

**Keywords:** neuroprotection, neural plasticity, amyloid beta - protein, cognitive function, hypoxia, ischemic stroke

## Abstract

Cognitive impairment is a common and disruptive outcome for stroke survivors, which is recognized to be notoriously difficult to treat. Previously, we have shown that low oxygen post-conditioning (LOPC) improves motor function and limits secondary neuronal loss in the thalamus after experimental stroke. There is also emerging evidence that LOPC may improve cognitive function post-stroke. In the current study we aimed to explore how exposure to LOPC may improve cognition post-stroke. Experimental stroke was induced using photothrombotic occlusion in adult, male C57BL/6 mice. At 72 h post-stroke animals were randomly assigned to either normal atmospheric air or to one of two low oxygen (11% O_2_) exposure groups (either 8 or 24 h/day for 14 days). Cognition was assessed during the treatment phase using a touchscreen based paired-associate learning assessment. At the end of treatment (17 days post-stroke) mice were euthanized and tissue was collected for subsequent histology and biochemical analysis. LOPC (both 8 and 24 h) enhanced learning and memory in the 2nd week post-stroke when compared with stroke animals exposed to atmospheric air. Additionally we observed LOPC was associated with lower levels of neuronal loss, the restoration of several vascular deficits, as well as a reduction in the severity of the amyloid-beta (Aβ) burden. These findings provide further insight into the pro-cognitive benefits of LOPC.

## Introduction

Cognitive impairment has been reported as one of the most debilitating side-effects of stroke, impacting up to 80% of survivors (1, 2). Problems with memory, learning, and attention can significantly impact a survivor's functional independence and several studies have reported that increased levels of cognitive impairment are associated with lower levels of self-reported quality of life (3). This situation has triggered a substantial effort both clinically and pre-clinically to develop effective strategies to improve cognitive function post-stroke (4, 5).

Currently, there are no approved therapeutic interventions for post-stroke cognitive impairment. Although promising, the use of individual pharmacological strategies e.g., donepezil and memantine, have a patchy record of success (2). Recent evidence has shown pro-cognitive effects of exogenously delivered growth hormone post-stroke (6–9). Another equally promising pro-cognitive therapy has been the use of intermittent exposure to a reduced oxygen environment (10, 11). This non-pharmacological approach has numerous advantages over current strategies including its well-characterized and acceptable safety-profile, relatively low cost, ease of delivery and scalability.

In the context of stroke, exposure to a low oxygen environment *prior* to induction of an ischemic event (up to and including 4 weeks prior) has been shown to produce robust neuroprotection (10). Whilst the exposure to low oxygen prior to an ischemic event is of interest, exposure post ischemic event is arguably a more translationally relevant time to evaluate. In this context, low oxygen post-conditioning (LOPC) has been demonstrated to exhibit significant therapeutic properties in the context of heart attack (12) and spinal cord injury (13) and there is a growing body of evidence to support its application post-stroke (14–19). Preclinical studies have shown LOPC to be neuroprotective (15), enhance neurogenesis (18, 19), and reduce the severity of secondary neuronal loss and atrophy in the thalamus (14, 17). LOPC has also been shown to improve motor function (15) and cognition (18, 19).

Despite evidence indicating the potential utility of LOPC as a therapy, the underlying mechanisms involved in driving the positive post-stroke exposure outcomes are relatively unknown. We have recently identified a number of mechanisms that correlate with post-stroke cognitive impairment including loss of neural tissue and vasculature, the accumulation of neurotoxic proteins including amyloid-beta (Aβ) (20), vascular leakage and aquaporin four (AQP4) depolarization (associated with effective clearance of neurotoxic proteins) (20). It is clear that post-stroke exposure to LOPC promotes neuronal survival and vascular growth (14, 15, 17), yet what remains unclear is whether LOPC improves other aspects of vascular function (i.e., AQP4 polarization), or whether these improvements can modulate the Aβ burden. Therefore, in this study we sought to consider whether LOPC influenced these mechanisms. We have also considered the impact of LOPC on several genes involved in regulating the expression of Aβ including production of Aβ [amyloid precursor protein (APP) (21); beta secretase enzyme−1 (BACE) (22); tumor necrosis factor α (TNFα) converting enzyme (TACE) (23)], transport of Aβ across the blood-brain barrier into the parenchyma [receptor for advanced glycation end products (RAGE) (24)], Aβ degrading enzymes [neprilysin (NEP) (25), endothelin-converting enzyme (ECE) (26) and insulin-degrading enzyme (IDE) (25)] and clearance of Aβ [low-density lipoprotein receptor-related protein 1 (LRP1) (27–29)].

## Materials and Methods

### Ethical Statement

Experiments were approved by the University of Newcastle Animal Care and Ethics Committee (A-2013-338), and conducted in accordance with the New South Wales Animal Research Act and the Australian Code for the Care and Use of Animals for Scientific Purposes. Animal research was undertaken in accordance with the ARRIVE guidelines (30).

### Animals

Male C57BL/6 mice (8 weeks old) were obtained from the Animal Services Unit at the University of Newcastle. Mice were maintained at 21 ± 1°C in a humidity controlled environment with food and water available *ad libitum*. Lighting was on a 12:12 h reverse light-dark cycle (lights on at 7 pm) with all procedures conducted in the dark phase. Mice were habituated for a minimum of 7 days prior to the start of the experiment.

### Experimental Design

A total of 128 mice were randomly allocated to one of the following four *cohorts*: [1] sham, [2] stroke, [3] stroke + 8 h LOPC/day (LOPC 8 h), or [4] stroke + 24 h LOPC/day (LOPC 24 h) *(n* = *32/cohort)*. Within each cohort mice were assigned to one of the following groups (a) behavioral testing, (b) fixed tissue analysis (immunohistochemistry), (c) western blotting, or (d) PCR analysis *(n* = *8/group*). Brain and blood samples were collected at day 17 post-stroke. Each outcome was analyzed by independent study team members blinded to the treatment conditions.

### Experimental Stroke and LOPC

Photothrombotic vascular occlusion was performed as previously described (31, 32). Briefly, animals were anesthetized using isoflurane (5% induction, ~2% maintenance) in 100% O_2_ followed by an intraperitoneal injection of 0.2 mL Rose Bengal dye (10 mg/ mL solution in sterile saline; Sigma-Aldrich, USA) or 0.2 mL vehicle (0.9% NaCl, Pfizer, Australia) for sham animals. After 8 min the skull was exposed and illuminated using a cold light source with a fibreoptic end of 4.5 mm diameter positioned 2.2 mm lateral and 0 mm posterior to bregma, targeting the left motor and somatosensory cortices for 15 min.

At 3 days post-stroke LOPC-treated mice were introduced into the low oxygen environment (11% O_2_). Low oxygen exposure was achieved using a customized ventilated cage racking system retrofitted to accept 11% oxygen, provided by a pressure swing adaptor based hypoxic generator (Mag20, Higher Peak, USA). Both CO_2_ and pressure were simultaneously monitored within the LOPC cages and remained at atmospheric levels ~350 ppm and normal sea level (101 kPa), respectively. LOPC treated mice, were exposed to 11% O_2_ for either 8 h/day (10 am to 6 pm) or 24 h/day for 2 weeks.

### Assessment of Cognitive Impairment

**Figure 1:**
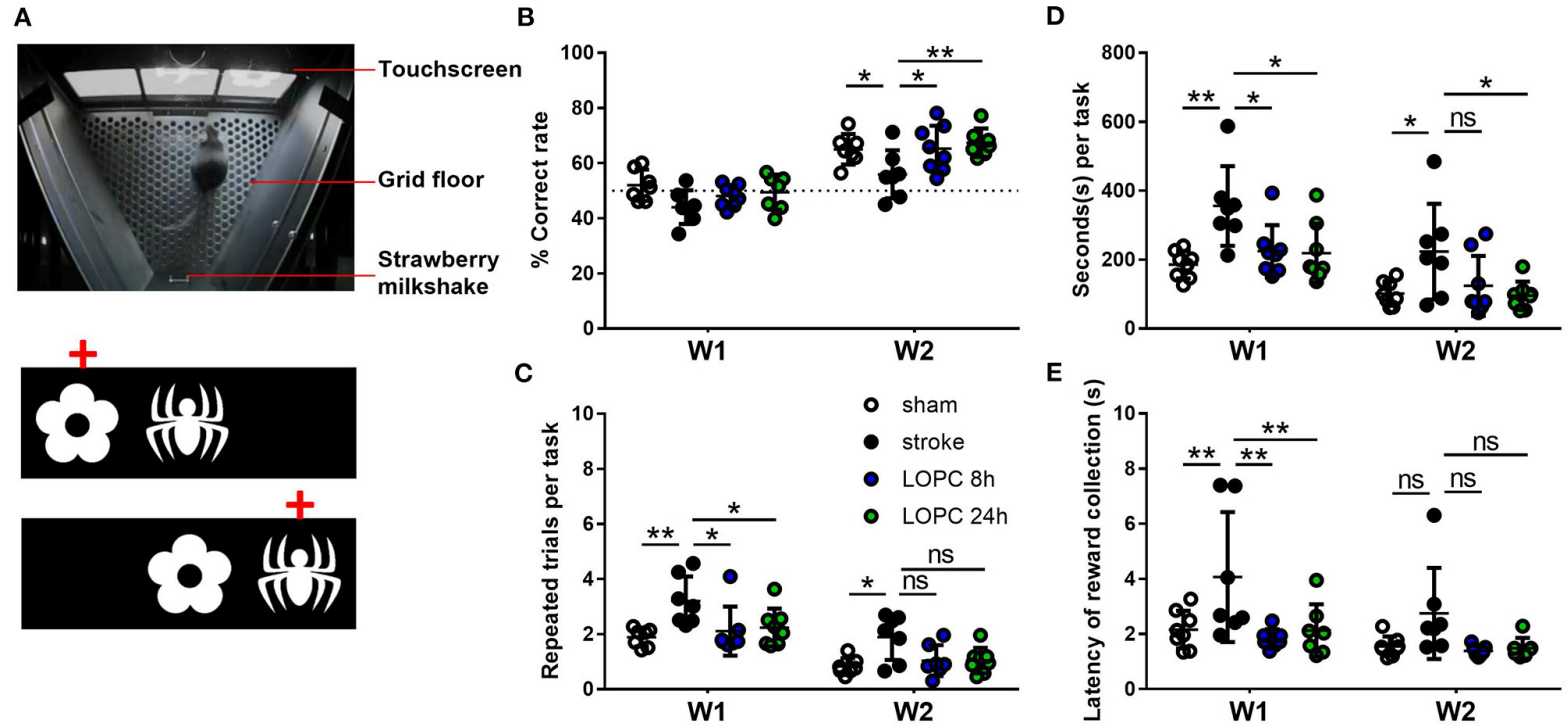
Illustration of the PAL task. **(A)** The Campden Instruments touchscreen chamber apparatus. To obtain the strawberry milkshake reward, animals were required to select the correct stimulus on the touchscreen. An illustration of the two different trial types and the correct location object pairing (red crosses) in PAL. Graphs show the performance of animals in each of the four groups (sham, stroke, LOPC 8 h and LOPC 24 h) in **(B)** % correct rate, **(C)** repeated trials per task, **(D)** seconds per task and **(E)** latency of reward collection in the first (W1) and 2nd week (W2) of treatment. Data is expressed as mean ± SD. ns, not significant; *p < 0.05, **p < 0.01 (two-way ANOVA, Tukey's multiple comparisons).

Associative memory was assessed in mice using the touchscreen platform for paired-associate learning (PAL) task. Touchscreen operant chambers (Campden Instruments Ltd., UK; Figure 1A) were used, and by their nature are inherently blinded. Individual animal performance was motivated using a liquid reward (strawberry milkshake; Paul's Milky Max). The task consists of two distinct phases: basic training, whereby the animal learns the association between making contact with the screen and the actual PAL task.

#### Habituation/Basic Training

Before stroke, animals were trained to touch the screen when it was illuminated in order to receive the liquid reward. Over 9 days all animals learnt to respond to screen illumination, with a minimum of 80% correct rate of response. Following general touchscreen training, mice underwent either experimental stroke or sham surgery.

#### PAL Task Learning

Three days post-surgery mice commenced the PAL task. In the task, three stimuli images (a flower, plane, and spider) are associated with a specific spatial location (left, center, right). In each trial, two images were displayed at the same time, one in the correct location and the other in an incorrect location (Figure 1A). All trials were mouse initiated and independent of the experimenter. If the animal touched the image in its correct location, a tone was triggered and a reward was provided (a correct trial was recorded). After reward collection the next trial was initiated. If the animal touched the incorrect image or the correct image in its incorrect location, it was punished by the absence of strawberry milkshake, no tone, and 5 s house light on (incorrect trial). Incorrect trials were separated by a 20 s inter-trial interval, and a repeated trial with presentation of the same stimuli was initiated. This process was repeated until either the mouse made the correct choice or 1 h had elapsed. The number of repeated trials (also termed the perseveration index) was recorded and was not counted in the total trials administered or the correct rate (i.e., % of correct trials). The time to finish each task was also recorded. Testing was terminated if the mouse successfully completed 36 trials or the testing session was 1 h in length.

### Haematocrit Assessment

Blood haematocrit levels were measured using the i-STAT system and CG8 cartridges (Abbott Point of Care).

### Tissue Processing

Mice were euthanized at 17 days post-stroke. For immunohistochemical analysis, animals were deeply anesthetized via a 0.2 mL intraperitoneal injection of sodium pentobarbitol (Lethabarb, Virbac, Australia, 325 mg/ mL) and transcardially perfused with ice cold saline for 2 min followed by ice cold 4% paraformaldehyde (pH 7.4) for 13 min. Brains were removed and post-fixed for 4 h in the same fixative and then transferred to a 12.5% sucrose solution in 0.1 M PBS for cryoprotection and storage. Serial coronal sections were sliced at 30 μm on a freezing microtome (−25°C; Leica, Australia). For western blot and PCR analysis animals were deeply anesthetized via an intraperitoneal injection of sodium pentobarbitol and transcardially perfused with ice cold 0.1% diethylpyrocarbonate in 0.9% saline for 2 min. Brains were dissected and rapidly frozen in −80°C isopentane. Sections were sliced at 200 μm using a cryostat microtome (−20°C, Leica, Australia). The peri-infarct territory (~2 mm^2^ surrounding infarct core) was punched using a 1 mm tissue punch. Samples were kept frozen at all times until protein and mRNA extraction.

### Histology and Immunohistochemistry

For immunoperoxidase labeling, free floating sections were immunostained as previously described (32) with one of the following primary antibodies: mouse anti-NeuN, mouse anti-GFAP, rabbit anti-Iba-1, rabbit anti-AQP4, biotinylated goat anti-IgG. Details on all antibodies have been provided in Supplementary Table 1. Pepsin antigen retrieval was performed on sections that were immunolabelled with rabbit anti-collagen IV using the method described by Franciosi et al. (33). Sections were rinsed with 0.1 M PBS and endogenous peroxidases were quenched in 0.1 M PBS containing 3% hydrogen peroxide. Non-specific binding was blocked with 3% normal horse serum. Sections were incubated in primary antibody with 2% normal horse serum for 48 h at 4°C and then were washed in 0.1 M PBS for 30 min and incubated with a biotinylated secondary antibody of corresponding species for 2 h at room temperature, rinsed, incubated in 0.1% extravadin peroxidase for 1 h, and then rinsed again. Immunolabelling was developed using a nickel-enhanced 3, 3'-diaminobenzidine (DAB) reaction. Tissues from the four treatment groups were stained simultaneously and the DAB reactions were developed for exactly the same length of time following the addition of glucose oxidase (1:1000). Negative control sections, in which no primary antibodies were added, were developed at the same time to confirm the specificity of labeling. After processing was completed sections were washed, mounted onto chrome alum-coated slides and cover-slipped.

### Image Acquisition, Tissue Loss, Cell Count, Thresholding, and AQP4 Polarization Analysis

**Figure 2:**
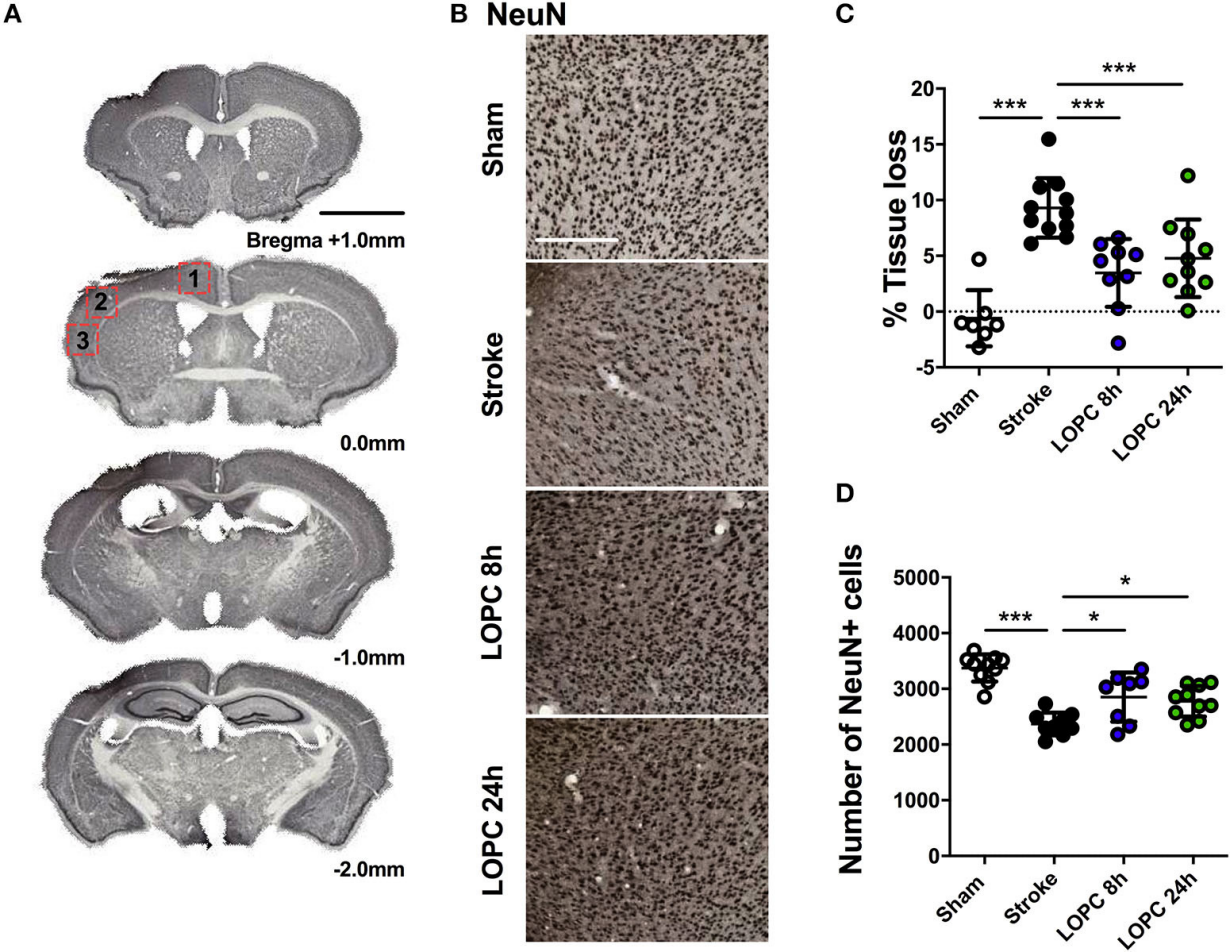
Illustration of the neural tissue loss. **(A)** The stroke sections from Bregma +1.0 mm to Bregma −2.0 mm. **(B)** Representative labeling for NeuN for the four groups: sham, stroke, LOPC 8 h and LOPC 24 h. **(C)** The graph shows that LOPC 8 h and LOPC 24 h animals had significantly lower % tissue loss compared to the stroke only animals. **(D)** The graph illustrates the total number of NeuN positive cells for the four groups. Multiple peri-infarct regions (red boxes) at Bregma 0.0 were included for neuronal cell counts. Data expressed as mean ± SD. *p < 0.05, ***p < 0.001 (one-way ANOVA, Tukey's multiple comparisons). White scale bar represents 300 μm and black scale bar represents 1 mm.

Images of DAB labeled tissue were acquired at 20× using Aperio AT2 (Leica, Germany). To estimate tissue loss within the infarcted hemisphere, the area of contralateral and ipsilateral hemispheres were measured across four sections (at Bregma levels +1.0, 0.0, −1.0, and −2.0 mm, Figure 2A) using ImageJ (34). The percentage of tissue loss was determined by the equation: [(average area of contralateral hemisphere – average area of ipsilateral hemisphere)/area of contralateral hemisphere] × 100. The quantitative analysis was undertaken specifically in the peri-infarct territory as defined by 0.1 mm from infarct, the region was 0.25 mm by 0.5 mm in size. Cumulative threshold analysis was performed using Matlab functions (35, 36). Firstly, for each of the acquired images, the number of pixels occurring at each of the pixel intensities was determined. The pixel intensity values are then rank ordered 0–255 along with the corresponding number of pixels that occur at each value. For the purposes of analysis, we calculated the percentage of cumulative threshold material for the range of pixel intensity values (Supplementary Figure 1). Pixel intensity level considered to be optimal for detecting genuine differences in immunoreactive signal was determined using ImageJ software to visualize thresholding of cropped regions at individual pixel intensities. This threshold level was used to investigate group differences for all labels. For NeuN, GFAP and Iba-1 positive cell counts, exhaustive manual cell counts were undertaken within the cropped regions (Bregma 0.0 mm, Figures 2A,B). The vessel digital reconstruction was performed as previously described (6). Collagen IV positive cells were isolated from the background using multi-level Otsu's thresholding method, which calculates the threshold that minimizes the interclass pixel intensity variance between groups. Using Matlab functions we determined percentage area covered by the labeling. AQP4 analysis involved quantifying the intensity of AQP4 labeling adjacent to the vessel lumen relative to that in the adjacent parenchyma (APQ4 vessel/parenchymal ratio).

### Protein Extraction and Western Blotting

Protein extraction and western blot were performed as previously described (31). Briefly, 15 μg of total tissue protein samples were electrophoresed 120 V for 2 h on Biorad Criterion TGC Stain-Free 4–20% gels. Gels were transferred to PVDF membranes (Biorad Immun-Blot PVDF Membrane, 0.2 μm pore size, binding capacity of 150–160 μg/cm^2^, #1620177) at 0.1A for 2 h in ice cold transfer buffer (25 mM Tris, 200 mM glycine, 20% methanol, pH 8.3). PVDF membranes were washed in Tris buffered saline with tween (TBST) (150 mM NaCl, 10 mM Tris, 0.075% Tween-20, pH 7.5) and blocked in 5% skim milk powder (SMP) in TBST for 1 h at 25°C. Membranes were incubated overnight with anti-Aβ antibody (D3D2N) #15126, Cell signaling Technology, 1:1000 in 1% SMP at 4°C, followed by horseradish peroxidase conjugated goat anti-mouse antibody, #170-6516, Biorad, 1:10,000 for 1 h at 25°C. In between each incubation step, membranes were washed in TBST. Membranes were visualized on an Amersham Imager 600 (GE Healthcare Life Sciences) using Luminata Forte Western blotting detection reagent (Merck Millipore). Aβ monomer (5 kDa) bands were lighter compared to the oligomers, and were visualized with longer exposure conditions. The membranes were then stripped and re-probed with anti-β-actin-peroxidase, AC-15 #A3854, Sigma-Aldrich, 1:50,000 for 1 h at 25°C. The housekeeping protein β-actin was used as a loading control to normalize the levels of protein detected. The density of the bands was measured using Amersham Imager 600 Analysis Software.

### mRNA Extraction and PCR

RNA was isolated using the Illustra RNAspin Kit (GE Healthcare, Cat# 25-0500-70) according to manufacturer's specifications from tissue punched from peri-infarct areas of frozen brain slices. cDNA was generated using SuperScript™ III First Strand Synthesis System for RT-PCR (Invitrogen, Cat# 18080-044) according to manufacturer's instructions on a GeneAmp PCR System 9700 instrument (Applied Biosystems). Quantitative RT-PCR was performed on an Applied Biosystems® 7500 (Applied Biosystems) or ViiA7 (Thermofisher) instruments using SensiFAST SYBR® Lo-ROX Master Mix (Bioline, Cat# BIO-94020). Genes of interest (Supplementary Table 2) were normalized to the housekeeping gene GAPDH and data are expressed as 2-ΔΔCt as fold change relative to sham.

### Statistical Analysis

All data is expressed as mean ± SD and was analyzed using GraphPad Prism version 6.01 (GraphPad Software, La Jolla, USA). Two-way ANOVA was used to determine whether there were time and treatment effects across groups in the PAL task. All other experiments used One-way ANOVA to determine whether there were any significant treatment effects across the groups. Additional Tukey multiple comparisons were used to analyse differences between the mean of each group and the mean of every other group. The significant differences shown on the graphs with asterisks (^*^) refer to the *post hoc* tests. All differences were considered to be significant at *p* < 0.05.

## Results

### LOPC Increases Haematocrit

To confirm the biological effect of the LOPC protocol, haematocrit assessment was performed on day 14 of LOPC treatment (17 days post-stroke). There was no significant difference between sham and stroke animals (0.38 ± 0.0006 vs. 0.38 ± 0.005, *p* = 0.97). The LOPC 8 h (0.48 ± 0.005) and LOPC 24 h (0.49 ± 0.008) animals had a significantly higher haematocrit than stroke only animals (*p* < 0.001 and *p* < 0.001, respectively).

### LOPC Ameliorates Cognitive Impairment After Stroke

#### Percentage of Correct Responses

There was a significant main effect for both treatment group (*F* = 118.3, *p* < 0.001) and time (*F* = 4.16, *p* < 0.05) (Figure 1B) on the percentage of correct responses. There were no statistically significant differences between groups in the 1st week post-stroke (*p* > 0.05). The sham and LOPC treated animals had significantly higher percentages of correct responses compared with the stroke group (sham, *p* < 0.05; LOPC 8 h, *p* < 0.05; LOPC 24 h, *p* < 0.01) in the 2nd week of testing.

#### PAL Metrics Used for Temporal Analysis

##### Number of Repeated Trials Per Task

We showed a significant main effect for group (*F* = 77.90, *p* < 0.001) and time (*F* = 6.36, *p* < 0.01) (Figure 1C) in the number of repeated trials per task. In the 1st week of active trials, sham and LOPC treated animals performed significantly less repeated trials when compared to the stroke animals (sham, *p* < 0.01; LOPC 8 h, *p* < 0.05; LOPC 24 h, *p* < 0.05). In the 2nd week of trials sham animals performed significantly fewer repeated trials compared with the stroke animals (*p* < 0.05), however there was no significant difference between either LOPC group and stroke animals (LOPC 8 h, *p* = 0.08; LOPC 24 h, *p* = 0.07).

##### Seconds Per Task

We found a significant main effect for group (*F* = 5.60, *p* < 0.01) and time (*F* = 84.59, *p* < 0.001) (Figure 1D) when analyzing the seconds taken to perform each task. In the 1st week, stroke animals took a significantly longer time per task when compared to the sham (*p* < 0.01), LOPC 8 h (*p* < 0.05) and LOPC 24 h (*p* < 0.05) groups. In the 2nd week of training stroke animals still took a significantly longer time per task when compared to sham (*p* < 0.05) and LOPC 24 h (*p* < 0.05) animals, however there was no significant difference between stroke and LOPC 8 h animals (*p* = 0.11).

##### Latency of Reward Collection

There was a significant main effect for group (*F* = 5.27, *p* < 0.01) and time (*F* = 14.03, *p* < 0.001) (Figure 1E). In the 1st week, stroke animals took significantly longer to collect rewards when compared to the sham (*p* < 0.01), LOPC 8 h (*p* < 0.01) and LOPC 24 h (*p* < 0.01) groups. There was no statistically significant deference between groups in the 2nd week.

### LOPC Reduces Tissue Loss and Neuron Loss After Stroke

The average volume of tissue loss was significantly higher in stroke animals compared to sham animals (*p* < 0.001). The average volume of tissue loss in the LOPC 8 h and LOPC 24 h animals was significantly smaller than what was observed in stroke only animals (*F* = 16.68, *p* < 0.001 and *p* < 0.001, respectively, Figure 2C). Stroke animals displayed significantly reduced numbers of NeuN positive cells in the peri-infarct territory compared to all other groups (*F* = 18.44; sham, *p* < 0.001; LOPC, 8 h *p* < 0.05; LOPC 24 h, *p* < 0.05. Figure 2D).

### LOPC Promotes Cerebrovascular Remodeling and Microglia Activation

**Figure 3:**
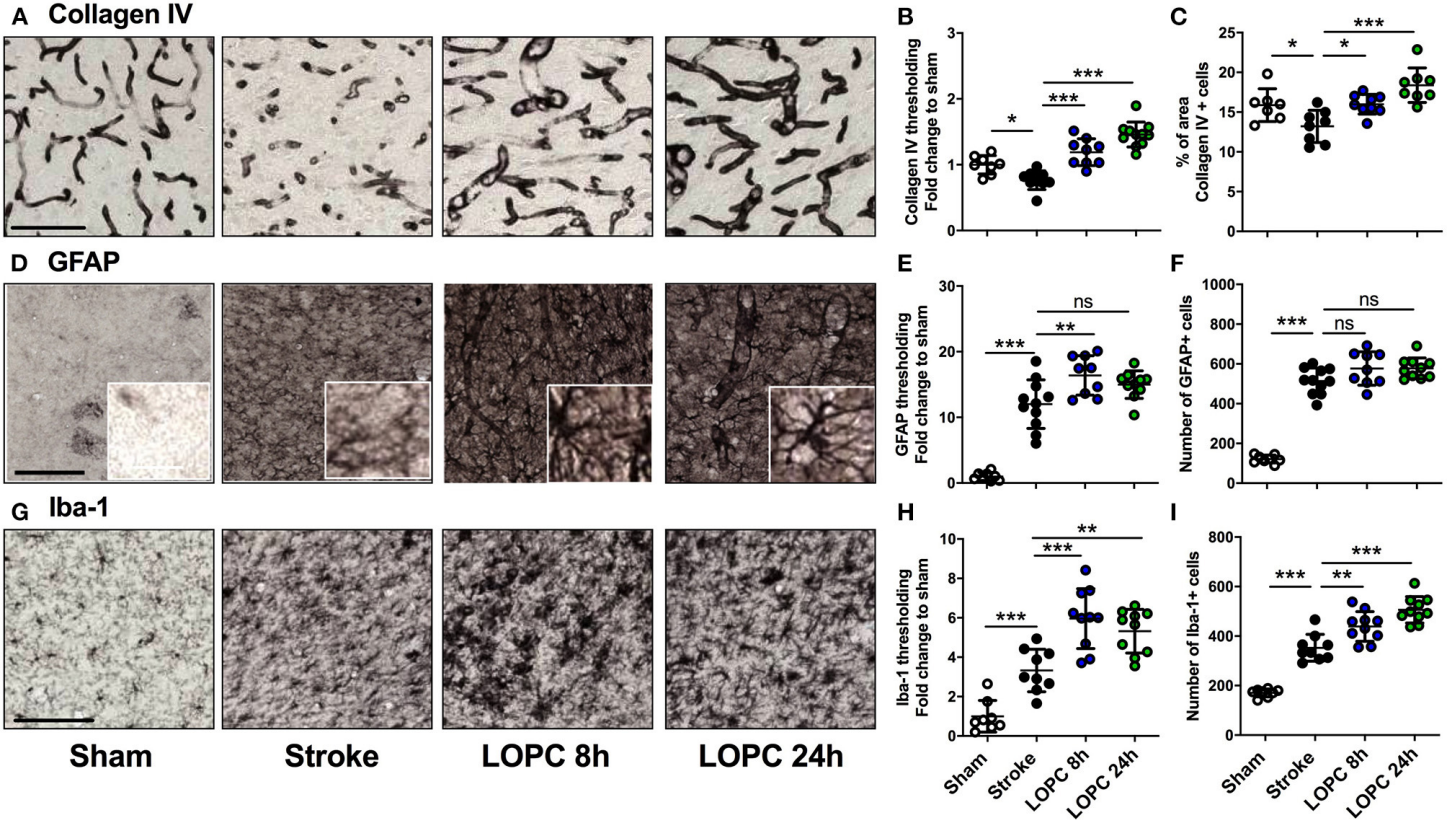
The effects of LOPC on vasculature and glial cells within the peri-infarct region. The four left panels in each row illustrate representative labeling for each marker investigated **(A)** Collagen IV, **(D)** GFAP (insets show astrocyte morphology at higher magnification) and **(G)** Iba-1 for the four groups: sham, stroke, LOPC 8 h, and LOPC 24 h. The first row of graphs **(B, E, H)** show quantification of the fold change of thresholded material for each of the markers. The second row of graphs show **(C)** percentage of area covered by Collagen IV positive cells, and number of **(F)** GFAP positive cells and **(I)** Iba-1 positive cells. Data is expressed as a fold change of mean ± SD for each group relative to the mean of the sham group. For cumulative threshold analysis refer to the Supplementary Figure 1. ns: not significant, *p < 0.05, **p < 0.01, ***p < 0.001 (ANOVA, Tukey's multiple comparisons). Black scale bars represent 100 μm and the white scale bar of inset represents 10 μm.

For thresholding analysis, the data for each group was expressed as a fold increase of the mean ± SD relative to the mean of the sham group (for cumulative threshold analysis see the Supplementary Figure 1). Stroke animals had significantly reduced Collagen IV immunoreactivity levels and percentage area covered by Collagen IV positive cells in the peri-infarct territory, compared to sham animals (*F* = 28.47, *p* < 0.05 and *F* = 10.02, *p* < 0.05, respectively; Figures 3A–C). Both LOPC 8 h and LOPC 24 h groups had significantly higher Collagen IV immunoreactivity levels compared with the stroke group (LOPC 8 h, *p* < 0.001; LOPC 24 h *p* < 0.001).

Stroke induced a significant increase in both GFAP and Iba-1 immunoreactivity relative to sham animals (*F* = 55.06, *p* < 0.001 and *F* = 31.72, *p* < 0.001, respectively; Figures 3D–I). This corresponded with an increase in the number of GFAP and Iba-1 positive cells in stroke animals compared to sham animals (*F* = 108.5, *p* < 0.001 and *F* = 72.68, *p* < 0.001, respectively). LOPC 8 h but not LOPC 24 h, exhibited modestly elevated thresholded immunoreactivity levels of GFAP compared to stroke animals (*p* < 0.01 and *p* > 0.05, respectively), despite no significant difference in the number of GFAP positive cells (*p* > 0.05 for both). LOPC 8 h and LOPC 24 h exhibited significantly increased thresholded immunoreactivity levels (*p* < 0.001 and *p* < 0.01, respectively), and Iba-1 positive cells (*p* < 0.01 and *p* < 0.001, respectively), compared with stroke animals.

### LOPC Improves Vascular Leakage and AQP4 Polarization After Stroke

**Figure 4:**
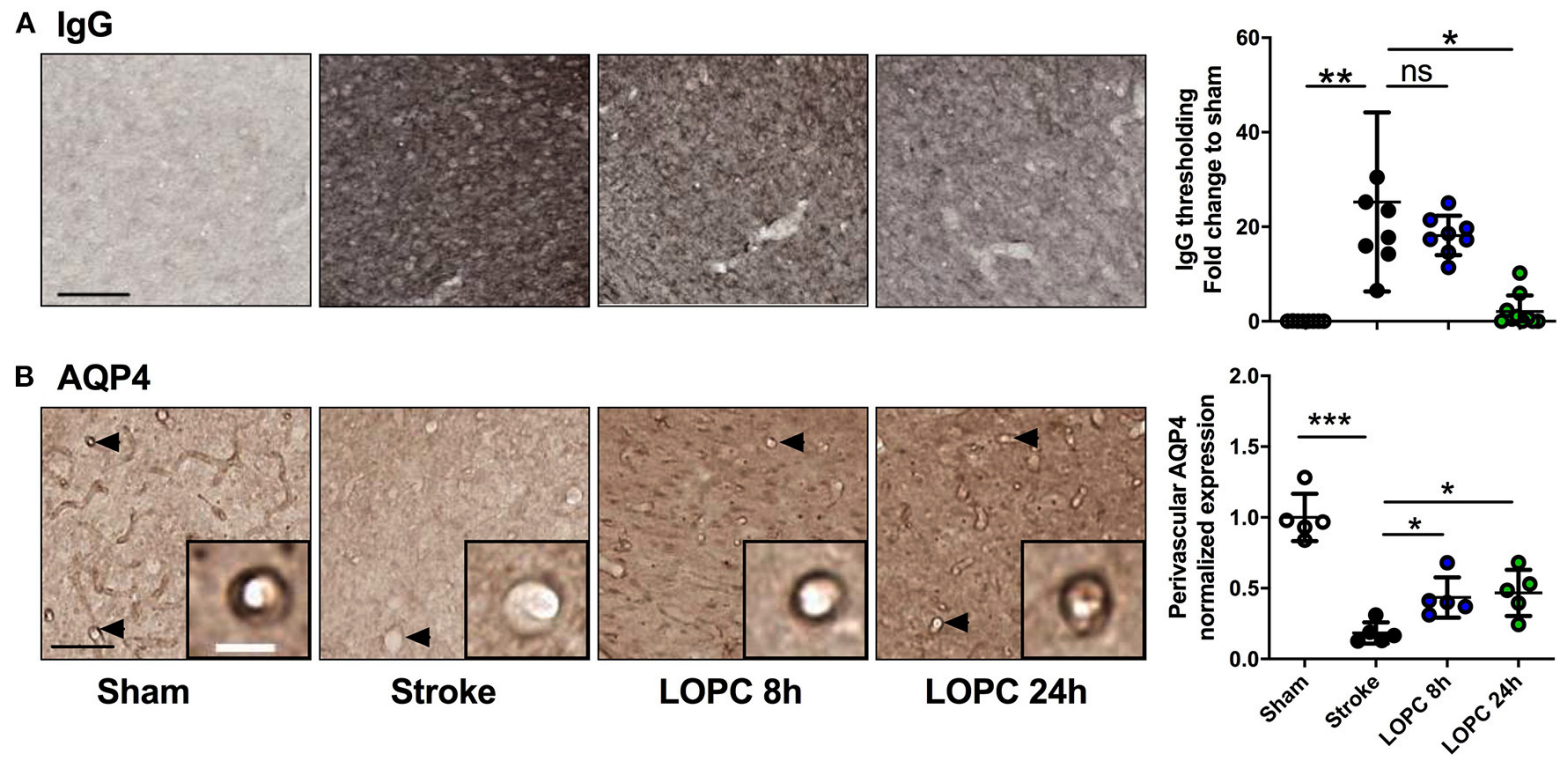
LOPC improves vascular leakage and AQP4 polarity within peri-infarct region following stroke. **(A)** Images illustrate representative labeling of IgG staining, an index of cerebrovascular leakage. Loss of blood-brain barrier integrity results in the infiltration of IgG into the tissue resulting in the diffuse staining pattern. The bar graph to the right shows the quantification of the fold change of thresholded material for IgG **(B)** Images illustrate representative labeling of AQP4. Insets show APQ4 polarity on vessels at high magnification. The dark color around the vessel represents AQP4 polarization on the endfeet of astrocytes (see arrows). The right bar graph illustrates the AQP4 polarization. Data expressed as a fold change of mean ± SD for each group relative to the mean of the sham group. ns: not significant, *p < 0.05, **p < 0.01 ***p < 0.001 (one-way ANOVA, Tukey's multiple comparisons). Black scale bar represents 100 μm and white scale bar of inset represents 10 μm.

Stroke-induced cerebrovascular leakage was assessed by IgG staining in the peri-infarct regions (Figure 4A). A significant increase in IgG (*F* = 13.87, *p* < 0.001) was present in stroke animals compared to sham animals. Vascular leakage was improved significantly with exposure to LOPC 24 h (*p* < 0.001), however there was no significant difference between LOPC 8 h and stroke only animals (*p* = 0.45).

AQP4 polarization was calculated as the ratio of AQP4 labeling on the vessel wall to that in the parenchymal tissue directly adjacent to the vessel (Figure 4B). AQP4 polarization toward the vessel walls was reduced significantly in peri-infarct regions of stroke compared to sham animals (*F* = 29.46, *p* < 0.001). This reduction was improved by both LOPC 8 h and LOPC 24 h (*p* < 0.05 and *p* < 0.05, respectively).

### LOPC Reduces Aβ Oligomer Accumulation After Stroke

**Figure 5:**
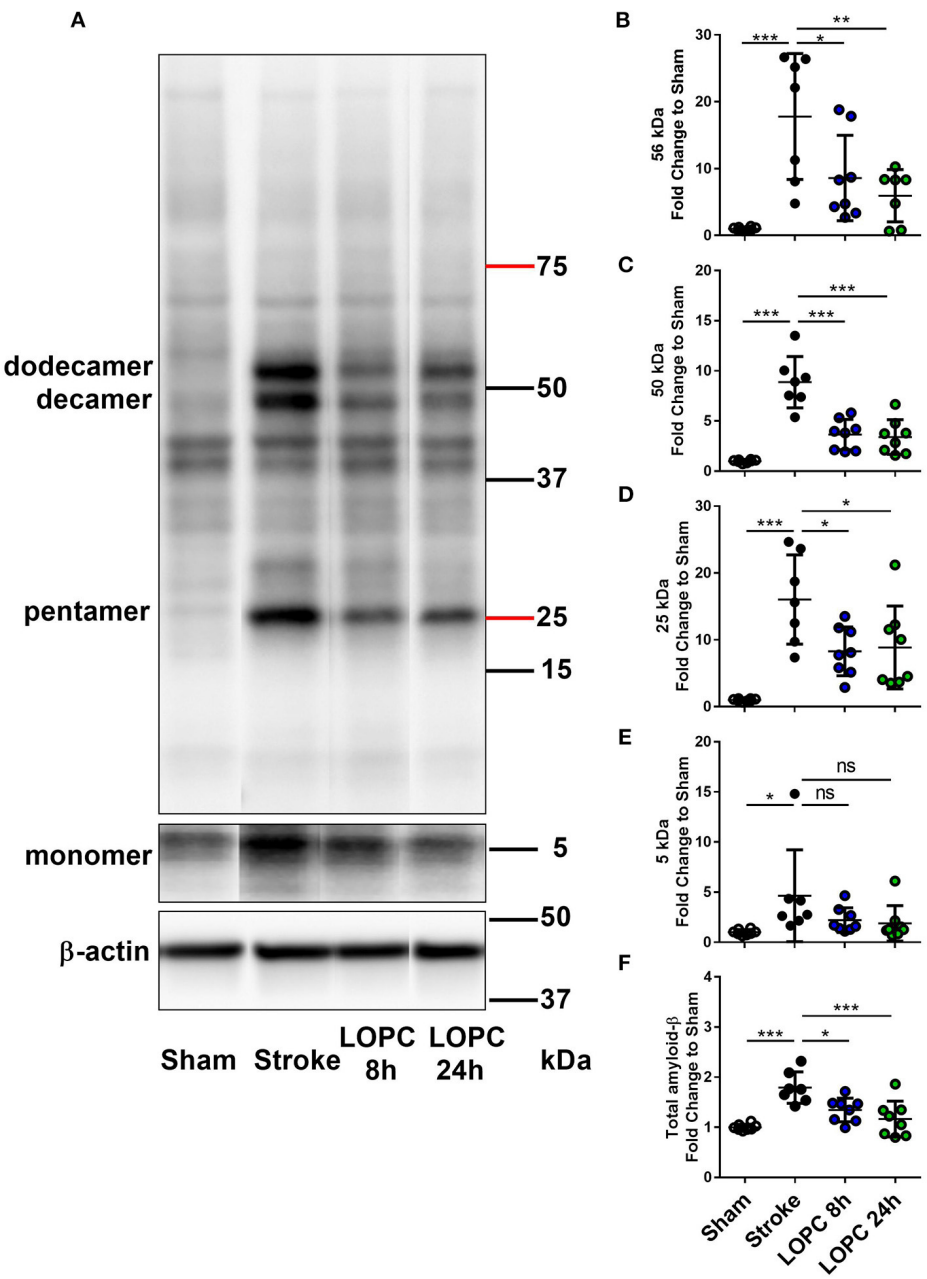
LOPC reduces Aβ in the peri-infarct territory after stroke. **(A)** A representative western blot of protein samples in peri-infarct territory from sham, stroke, LOPC 8 h, and LOPC 24 h animals. Bands were detected using D3D2N anti-Aβ antibody. Loading controls were performed by analysis of β-actin. For raw western blot data refer to the Supplementary Figure 2. The graphs at the right are quantification of Aβ oligomers at **(B)** 56 kDa (dodecamer), **(C)** 50 kDa (decamer), **(D)** 25 kDa (pentamer), **(E)** 5 kDa (monomer) and **(F)** total Aβ (5–200 kDa) deposition. Data is expressed as a fold change of mean ± SD for each group relative to the mean of the sham group. ns: not significant, *p < 0.05, **p < 0.01 ***p < 0.001 (one-way ANOVA, Tukey's multiple comparisons).

Data for all groups are expressed as a fold increase of the mean ± SD for each group relative to the mean of the sham group. All Aβ oligomers showed similar patterns (Figure 5; raw data is shown in Supplementary Figure 2). Specifically, at 56 kDa, 50 kDa, 25 kDa, and total Aβ levels, were elevated in the stroke group relative to sham animals (*F* = 10.44, *p* < 0.001; *F* = 28.60, *p* < 0.001; *F* = 12.10, *p* < 0.001; and *F* = 12.14, *p* < 0.001, respectively). Both the LOPC 8 h and LOPC 24 h displayed lower levels of oligomerization relative to the stroke alone condition (LOPC 8 h: *p* < 0.05, *p* < 0.001, *p* < 0.05, *p* < 0.05, and LOPC 24 h: *p* < 0.01, *p* < 0.001, *p* < 0.05, *p* < 0.001, respectively). Regarding the 5 kDa oligomer, stroke induced a significant increase relative to sham animals (*F* = 3.01, *p* < 0.05), but there was no significant difference compared to LOPC 8 h and LOPC 24 h (*p* = 0.23 and *p* = 0.15, respectively).

### LOPC Alters APP and BACE mRNA Expression

**Figure 6:**
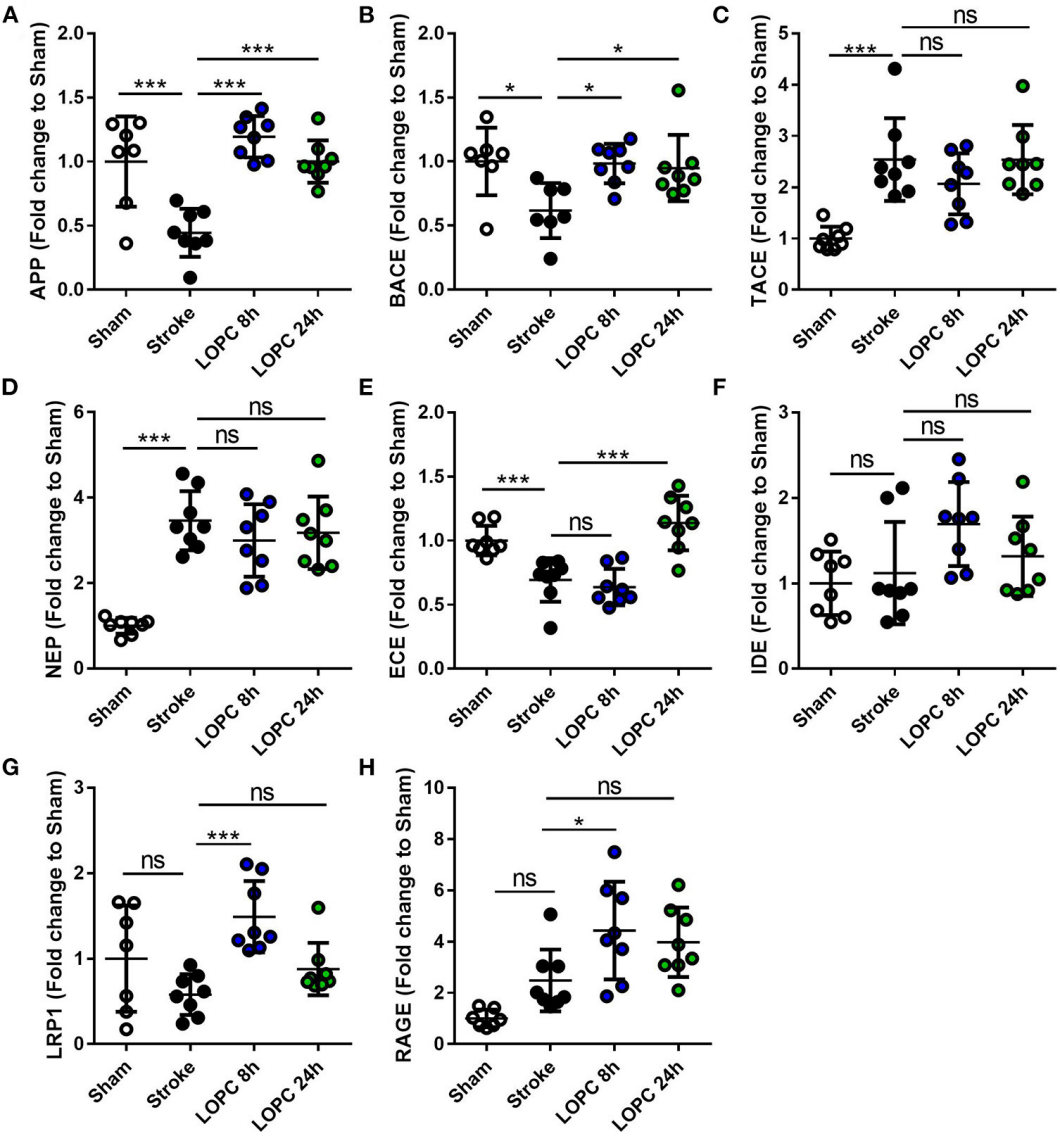
The expression levels of genes involved in the generation, degradation and export of Aβ. The expression of **(A)** amyloid precursor protein, APP; **(B)** beta-secretase, BACE; **(C)** TNFα converting enzyme, TACE; **(D)** neprilysin, NEP; **(E)** endothelin-converting enzyme, ECE; **(F)** insulin-degrading enzyme, IDE; **(G)** low-density lipoprotein receptor–related protein-1, LRP1; and **(H)** receptor for advanced glycation end products, RAGE. Data is expressed as a fold change of mean±SD for each group relative to the mean of the sham group. ns: not significant, *p < 0.05, ***p < 0.001 (one-way ANOVA, Tukey's multiple comparisons).

Stroke animals exhibited a significant decrease in the expression of APP and BACE mRNA levels relative to sham animals (*F* = 16.56, *p* < 0.001 and *F* = 4.61, *p* < 0.05, respectively). This reduction of APP and BACE mRNA levels was reversed by both LOPC 8 h and LOPC 24 h. Stroke alone induced a significant increase in the expression of TACE and NEP mRNA levels relative to sham animals (*F* = 11.13, *p* < 0.001, and *F* = 20.62, *p* < 0.001). However, stroke alone induced a significant decrease in the expression of ECE mRNA levels relative to sham animals (*F* = 17.14, *p* < 0.001). LOPC 8 h modestly elevated LRP1 and RAGE mRNA levels (*p* < 0.001 and *p* < 0.05, respectively), and LOPC 24 h increased ECE mRNA levels (*p* < 0.001), relative to stroke only animals. See Figure 6.

## Discussion

LOPC represents an interesting future therapeutic strategy for improving cognitive function after stroke. While the published literature so far is modest in size, both preclinical (14–19) and small-scale clinical studies (11) have produced promising results. Both our own group and others indicated that stroke is associated with the accumulation of what may be best considered as neurotoxic waste products, such as Aβ and others (20, 31, 37, 38). Rather than considering these waste products to be directly pathological, we have been pursuing a line of thought that cognitive impairment may be, in part, underpinned by the inability of the brain, post-ischemic injury, to effectively rid itself of waste products. Given the prior work indicating the ability of LOPC to improve cognition, we were intrigued by the possibility that LOPC may be associated with evidence of improved waste product removal. As a comprehensive investigation of all waste products was impractical, we decided to principally focus our attention on changes in the accumulation of Aβ oligomers.

The study design that we employed was relatively straight forward with animals undergoing experimental stroke and either being exposed to LOPC or not. As was predicted and consistent with earlier reports, we found that both the 8 and 24 h LOPC exposure regimens improved cognition as indexed by performance on the PAL task. Amongst the significant new findings, we identified robust evidence suggesting that LOPC, in addition to triggering vessel growth, reduced vascular leakage, as indexed via IgG deposition proximal to cerebral vessels. Aligning well with these vascular improvements we further discovered that LOPC triggers a re-polarization of AQP4, a key protein involved in water movement through the brain, toward its regular location, proximal to the vessel surface. Finally, we observed quite marked reductions in Aβ oligomers in the LOPC exposed groups. While this evidence is too preliminary to confirm the ability of LOPC to improve vascular function and improve waste clearance, it is suggestive that further exploration of this line of investigation would be well-justified.

Clinically, the investigation of changes in cognition would normally involve participants undertaking a comprehensive multi-domain neuropsychological test battery. While performing such an approach in the rodent would absolutely produce highly translational results, the unfortunate reality is that due to fundamental differences in the speed of knowledge acquisition, the amount of training time required in rodents to perform a similar battery is not practical. As such, it is frequently the case that studies using rodents have tended toward using single-domain assessments. Historically, a strong preference was placed on reasonably rapid assessments, such as the novel object recognition task, or more comprehensive assessments such as the Morris water maze. Over the past decade, considerable concern has been raised in the scientific literature about the appropriateness of these approaches due to notorious levels of variability and the use of fear and stress to motivate performance (39–41). This concern has produced a migration in the field of discovery research toward the use of touch-screen based approaches (41, 42). While representing a significantly more expensive investment both in terms of capital infrastructure and human resources, the approach is widely considered to be superior in terms of consistency in reproducibility (42). It was largely for these reasons that we adopted our method of assessment in the current study.

In using the touchscreen based assessments we decided to utilize the PAL task. The task evaluates visuospatial learning and memory (41, 42). Specifically, the rodent is rewarded for responding to a particular visual symbol, only if the symbol is presented in a particular location. If the rewarded cue is presented in the incorrect location and a selection is made then an incorrect choice is logged. Our experience and the experience of others has indicated that it takes ~2 weeks for the animals to develop an understanding of the associations involved in order to accurately respond (6). Our decision to use the task was motivated on the basis that stroke survivors demonstrate robust impairments in PAL learning (1, 43), a finding that is also in line with other studies indicating the existence of verbal associate learning deficits (44). In the current study, we observed that mice exposed to stroke only produced a significantly higher rate of errors compared to sham animals in the 2nd week of trials. In contrast, animals exposed to LOPC showed improvements in the number of correct choices made over the stroke group. This evidence is quite consistent with other earlier reports demonstrating the ability of LOPC to improve cognition (18–20). The result relating to the number of correct choices was also reinforced by a second metric of performance, the number of repeat trials undertaken to achieve a successful outcome. This secondary metric largely followed the trend observed in the number of correct choices made data set.

The most obvious explanation for the pro-cognitive effect of LOPC is that the intervention is neuroprotective (45). Consistent with our previous findings (15), we observed a neuroprotective effect of LOPC using two indices. Firstly, we identified that mice exposed to LOPC exhibited a reduced area of brain tissue loss relative to the stroke group, and secondly we demonstrated an increase in the number of NeuN+ neurons. This result could be accounted for in a number of ways. Firstly, LOPC may delay neuronal death. Further insight could be gained through the use of TUNEL staining to identify cells undergoing apoptosis. However, we have previously shown this neuroprotective effect to extend out to 15 days post-LOPC treatment and therefore think it unlikely that LOPC simply delays neuronal death (15). Based on this, a more plausible explanation is that LOPC may reduce cell death and/or it may stimulate neurogenesis [as has been previously documented in the hippocampus (18, 19)]. While it was not possible in the current study (due to sample limitations) to consider makers of neurogenesis this would be a particularly interesting avenue for future investigation and could be investigated using bromodeoxyuridine labeling.

LOPC was observed to exert wide ranging vascular and neurovascular effects. Of particular importance we observed that the haematocrit (the proportion of erythrocytes in the blood) was significantly elevated in LOPC exposed animals. Elevated haematocrit is a well-recognized physiological adaption to hypoxia (46, 47), thus effectively providing a positive control for the intervention. As expected we observed that LOPC resulted in enhanced vessel density, with levels of Collagen-IV being markedly higher in LOPC exposed groups (4, 10, 48). We also identified that LOPC significantly reduced vascular leakage, as indexed by peri-vascular IgG labeling. Typically, no labeling for IgG is identifiable around vessels within the brain, however, this was significantly increased in animals that had a stroke and reduced by LOPC exposure. Furthermore, the slight increase in GFAP immunoreactivity as well as the restoration of AQP4 polarization observed in this study suggests that LOPC may also exert effects over astrocytes. AQP4 is recognized to be expressed in the end-feet of astrocytes that make contact with the vessel wall. The protein itself is considered to play an integral role in facilitating water transport into the parenchyma (49). We observed that polarization of AQP4 was significantly disrupted by stroke, with nearly a complete loss of polarization of AQP4 in the region considered. Further, we observed LOPC to largely restore AQP4 polarization. Taking into consideration the fact that LOPC produced more vessels (or reduced the loss of vessels), that the vessels present in LOPC exposed animals exhibited less leakage of IgG and that AQP4 polarization was present we would propose that the LOPC restores many of the critical elements required for the removal of waste products from the brain.

LOPC treated animals displayed a mild stimulation of microglia, as evidenced by an enhanced level of Iba-1 expression. This modest enhancement is particularly interesting given the recent work demonstrating the essential role of microglia in mediating vascular repair (33, 50, 51). Further characterizing microglial engagement with vascular repair in the context of LOPC represents a promising area of future exploration. It is important to note that although we observed increased activation of microglia and astrocytes, we did not specifically investigate the activation states. We have previously shown that LOPC decreases CD45 (15), CD68 (15) [markers of M1 activation (52)], and that LOPC moderately dampens the neuroinflammatory tone and increases microglial ramification suggesting a less inflammatory state compared with non-treated animals (15). However, further investigation into M2 markers [e.g., CD206 (52)] would have helped elucidate whether the changes we observed in this study were beneficial or harmful. Further, characterization of the astrocyte phenotype using markers e.g., C3d and S100A10 [markers of A1 and A2 states (53)] is also an important future direction. This is a clear limitation in our study.

We further observed that LOPC promoted the removal of Aβ, a neurotoxic protein which is deposited at higher levels after stroke, and is well-recognized to disrupt neuronal function (18, 51, 54, 55). To our knowledge, this is the first report to demonstrate that LOPC is capable of inducing robust decreases in total levels of amyloid and reducing aggregation of soluble Aβ oligomers post-stroke. In line with published findings, we identified that animals exhibited greater levels of the soluble oligomers of Aβ post-stroke (20, 54). These levels were significantly reduced in the animals exposed to LOPC. Further investigation showed specific reductions in 25, 50, and 56 kDa oligomers in LOPC treated animals relative to the stroke alone. Soluble Aβ oligomers have been linked with cognitive decline within the context of neurodegenerative diseases including stroke (20, 45, 56–58). Furthermore, multiple studies in the context of Alzheimer's disease have suggested that soluble Aβ oligomers, and not plaques, are linked with cognitive decline (45, 56, 58). Thus, we speculate that the reduction in Aβ seen in this study may be linked with the improvement in cognition. However, we believe it is also important to recognize that we have only demonstrated an associative relationship between LOPC, reduced Aβ and improved cognition. While Aβ is known to interfere with synaptic function, it may be possible that LOPC exerts is positive effects on cognition by improving clearance of waste proteins and molecules from the brain (or even improved vascularisation alone). If this were the case the LOPC induced changes in Aβ may be better considered as an indicator of improved vascular function and/or clearance processes and not causatively responsible for improved cognition. The significance of the results presented in the current study is that they should allow for these emergent questions to be considered with precision by future studies.

There are several possible explanations for the ability of LOPC to reduce the Aβ burden post-stroke. Firstly, changes in Aβ expression may result from changes in the rate of production of APP and its conversion into Aβ (via alpha- and beta-secretase). When investigating this, we observed that stroke induced disturbances in the expression levels of the APP and BACE mRNA and that LOPC restored these levels. Importantly, the expression levels of APP and BACE in the LOPC exposed animals did not differ from those in the sham group. As such, LOPC appears to produce a more “normal” expression phenotype for APP and BACE. The extent of intracellular ingestion of Aβ and the actions of degrading enzymes (NEP, ECE and IDE) may also influence expression of Aβ. However, we did not find any evidence to suggest that LOPC altered the expression of other enzymes involved in Aβ formation or digestion including TACE, NEP, or IDE. Alternatively, it may be influenced by the availability of vascular bound transporters LRP-1 and RAGE. Modest elevations were observed in LOPC 8 h exposed animals over stroke alone in mRNA expression of LRP-1 and RAGE products. The elevated expression of LRP-1, which has a well-documented role in the export of Aβ (27–29), is consistent with our findings of reduced Aβ levels. The increase in RAGE may be best interpreted by considering the role of RAGE in facilitating microglial chemotaxis toward Aβ (59) and/or phagocytosis (60, 61).

Together the results from the current study suggest that LOPC improves Aβ loading via a constellation of changes in the processing and removal of Aβ. Coupled with the improvements in the neurovascular unit, we hypothesize that the reduced levels of Aβ are likely the result of improved vascular density and improved vascular transportation. However, further experimentation here is required, ideally using the intracranial delivery of tagged-Aβ that can be tracked in real-time.

LOPC is emerging as a promising therapeutic intervention in experimental stroke. A key finding of this study was that treatment with only 8 h of LOPC was similar to treatment with 24 h of LOPC. A recent study by Wang et al. (11) has shown cognitive improvement by exposing patients with mild cognitive impairment to intermittent hypoxia (8 × 5 min cycles for 3 × weekly at 10% O_2_) (11), suggesting that LOPC is effective at improving cognition in patients with much shorter exposure times. Future studies will be designed to determine the minimum time required for LOPC treatment post-stroke.

One of the principle advantages associated with reduced oxygen exposure interventions is that the approach appears to trigger system-wide compensatory adaptions. The findings from this study support the potential of LOPC for promoting recovery post-stroke. We have identified that exposure to a low oxygen environment for 2 weeks following stroke, beginning 3 days post-infarction, is sufficient to improve cognition, reduce neuronal loss, restore several vascular deficits, as well as reduce the severity of the Aβ burden. Stroke is known to trigger cognitive impairment and under certain circumstances trigger the emergence of dementia-like symptoms. A therapeutic strategy to reduce Aβ and improve cognitive performance is highly desirable. As several human specific technologies already exist for controlled oxygen exposure, it is not inconceivable that future safety trials for this promising pro-cognitive intervention could be undertaken rapidly.

## Data Availability Statement

The original contributions presented in the study are included in the article/Supplementary Material, further inquiries can be directed to the corresponding author/s.

## Ethics Statement

The animal study was reviewed and approved by Animal Care and Ethics Committee, University of Newcastle, NSW, Australia.

## Author Contributions

ZZ, MN, and FW designed the experiment. ZZ performed the majority of the experiments. LO undertook all western blotting analyses and prepared the results for these data. GP undertook all mRNA analyses and prepared the results for these data. SS, KW, MI, MK, and CT assisted in the experiments. SJ designed and prepared program for the image processing. ZZ, LO, GP, OPO, SJ, MN, and FW analyzed the data and interpreted the results. ZZ, RH, LO, and FW wrote the paper. RH, LO, GP, SS, KW, MI, MK, CT, OPO, SJ, and MN revised all drafts and the manuscript. All authors contributed to the article and approved the submitted version.

## Conflict of Interest

The authors declare that the research was conducted in the absence of any commercial or financial relationships that could be construed as a potential conflict of interest.
