# Supplementary material for: Exploring How Low Oxygen Post Conditioning Improves Stroke-Induced Cognitive Impairment: A Consideration of Amyloid-Beta Loading and Other Mechanisms
Source: Front Neurol. 2021 Mar 24;12:585189. doi: 10.3389/fneur.2021.585189 (PMC8024636; doi:10.3389/fneur.2021.585189)
Supplement: Supplementary file 1 [file Data_Sheet_1.docx]

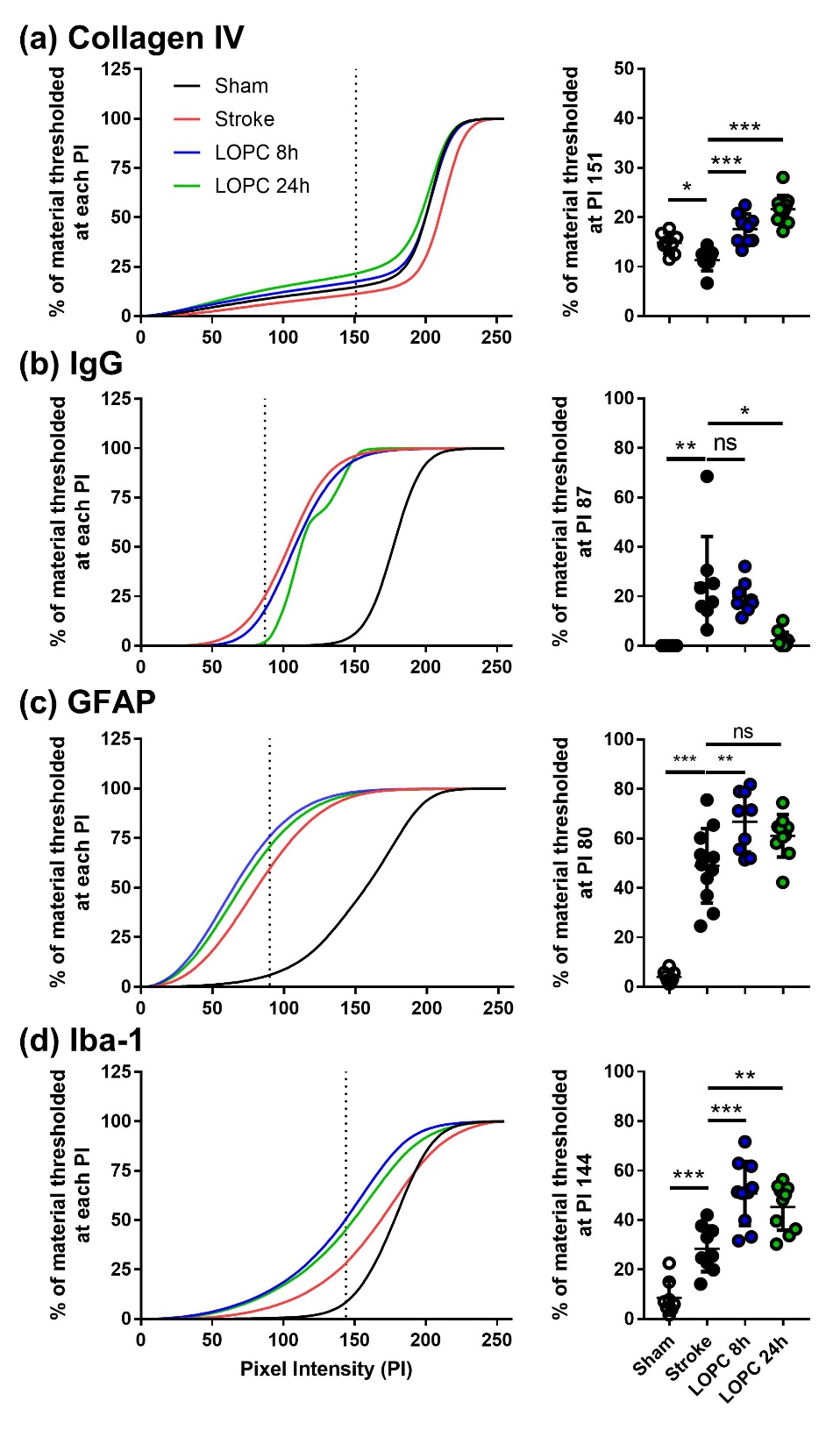


**Supplementary figure 1.** **Cumulative threshold analysis.** Left panels show the percentage of material thresholded of (**a**) Collagen IV, (**b**) IgG, (**b**) GFAP and (**d**) Iba-1 immunolabels at different levels of pixel intensity (PI). The dotted lines are the PI levels considered to be optimal for detecting genuine differences in immunoreactive signal. The right most panel illustrates quantification of the change % of thresholded material for each of the immunolabels at selected PI. Data for left panels are presented as mean and data for right panels are presented as mean±SD. ns: not significant, **p*<0.05, ***p*<0.01, ****p*<0.001 (ANOVA followed by Tukey’s multiple comparisons).


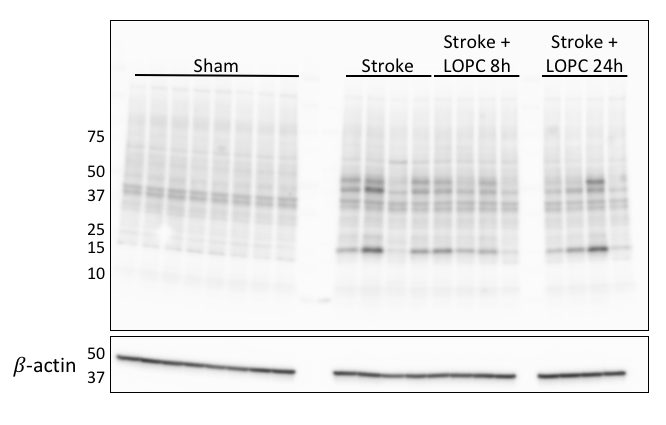


**Supplementary figure 2.** **Raw data: Amyloid-** **𝛽 expression in stroke mice vs sham, LOPC 8h and LOPC 24h.** The top panel shows a representative western blot of protein samples in the peri-infarct territory from sham, stroke, LOPC 8h and LOPC 24h animals. Bands were detected using D3D2N anti-Aβ antibody. Loading controls were performed by analysis of β-actin (bottom panel).

**Supplementary table 1.** List of antibodies used for western blot and immunohistochemistry.

| **Target** | **Sources of antibodies** | **Application** | **Dilution** |
| --- | --- | --- | --- |
| **Collagen IV** | Abcam, rabbit anti-collagen IV, ab6586 | IHC | 1:1000 |
| **GFAP** | Sigma, mouse anti-GFAP, G3893 | IHC | 1:1500 |
| **Iba-1** | WAKO, rabbit anti-Iba-1, 019-19741 | IHC | 1:1000 |
| **NeuN** | Millipore, mouse anti-NeuN, MAB377 | IHC | 1:500 |
| **AQP4** | Millipore, rabbit anti- AQP4, AB3594 | IHC | 1:1000 |
| **IgG** | Abcam, biotinylated goat anti-mouse IgG, ab64255 | IHC | 1:500 |
| **amyloid β** | Cell Signaling Technology, anti-amyloid β (D3D2N), #15126 | WB | 1:1000 |
| **β-actin** | Sigma-Aldrich, Monoclonal anti-β-actin-HRP, A3854 | WB | 1:50000 |
| **Rabbit IgG** | Jackson ImmunoReseach, goat anti-rabbit-biotin, #111-065-003 | IHC | 1:500 |
| **Mouse IgG** | Biorad, anti-Mouse-HRP antibody, #170-6516 | WB | 1:10000 |
|  | Jackson ImmunoReseach, goat anti-mouse-biotin, #115-065-003 | IHC | 1:500 |

WB, western blot; IHC, immunohistochemistry.

**Supplementary table 2.** PCR primer sequences.

| **Genes** | **Forward** | **Reverse** |
| --- | --- | --- |
| **APP** | CCCACGACAGCAGCCAG | GCTCTGCCTCTTCCCATTC |
| **BACE1** | GCTTTGTGGAGATGGTGGAC | AGGATGTTGAGCGTCTGTGG |
| **TACE** | CATCGTTGGGTCTGTTCTGG | AGGGATTCATACTGCTTGTC |
| **NEP** | CTGGAGGTCAATGGGAAGTC | TCGGCTGAGGCTGCTTAC |
| **ECE** | TCCTTCGCTGCCCTCCT | GGACAACATCAAAAGACCCAC |
| **IDE** | AGTTCCCTGAGCACCCTTTC | ACCCAGCCCTTTGATTTGAG |
| **LRP1** | AGACTATCAGGGCGGCAAG | CAAACACGGACACGGAGAAC |
| **RAGE** | CCGATGGCAAAGAAACACTC | GCAGGAGAAGGTAGGATGGG |

APP, amyloid precursor protein; BACE1, Beta-secretase 1; TACE, TNFα converting enzyme; NEP, neprilysin; ECE, endothelin-converting enzymes; IDE, insulin-degrading enzyme; LRP1, low-density lipoprotein receptor-related protein; RAGE, receptor for advanced glycation end products.
